# Engaging Patients and Caregivers in Co-Design of a Patient Portal-Based Deprescribing Intervention

**DOI:** 10.1093/geroni/igaf122.1649

**Published:** 2025-12-31

**Authors:** Tobie Taylor-McPhail, Kathy Gleason, Andrea Daddato, Rosalphie Quiles, Jennifer Portz, Elizabeth Bayliss, Ariel Green

**Affiliations:** Kaiser Permanente Colorado, Aurora, Colorado, United States; Kaiser Permanente Colorado, Aurora, Colorado, United States; Kaiser Permanente Colorado, Aurora, Colorado, United States; Johns Hopkins University School of Medicine, Baltimore, Maryland, United States; University of Colorado School of Medicine, Aurora, Colorado, United States; Kaiser Permanente Colorado, Aurora, Colorado, United States; Johns Hopkins University School of Medicine, Baltimore, Maryland, United States

## Abstract

Successful deprescribing requires older adult and caregiver engagement, yet few deprescribing interventions have been co-designed with people with cognitive impairment and caregivers. We convened an advisory panel with 19 older adults with cognitive impairment and their caregivers from across the United States in four listening sessions (June 2024–March 2025) to refine the language and appearance of a patient portal-based deprescribing intervention involving an outreach message and educational video. We co-designed messaging, video content, and optimal patient/caregiver preparation for a deprescribing conversation. Each session followed a discussion guide, and participants received session summaries and incentive gift cards. The collaborative design process provided key insights, emphasizing: 1) reassure viewers the older adult’s prescribing provider will be involved in the deprescribing process, 2) highlight the caregiver’s role in the deprescribing conversation, and 3) describe the role of clinical pharmacists in clinical care. Based on feedback, we simplified video messaging, focused on accurate character portrayals, and added action steps. While most found the video motivating, they preferred discussing deprescribing during visits with prescribing clinicians rather than scheduling separate appointments. As patient portals become more universal, designing interventions that resonate with older adults and caregivers is increasingly important. The intervention will be piloted as a next step to inform a pragmatic deprescribing intervention. Engaging an advisory board with lived experience in the co-design of a portal-based deprescribing intervention for people with cognitive impairment led to important modifications of the message and video.

